# Radical Chemoradiotherapy for Urethral Squamous Cell Carcinoma: Two Case Reports and a Review of the Literature

**DOI:** 10.1155/2013/194690

**Published:** 2013-05-07

**Authors:** H. Coop, L. Pettit, C. Boon, P. Ramachandra

**Affiliations:** Deansley Centre, Royal Wolverhampton Hospital, Wednesfield Road, Wolverhampton WV10 0QP, UK

## Abstract

Primary urethral squamous cell carcinoma is rare. Its management is particularly challenging owing to the paucity of evidence from randomised trials to inform practice. We report two male and female cases of squamous cell carcinoma of the urethra, which were treated with concomitant cisplatin and radiotherapy. These cases add to the body of case reports that have shown benefit for concomitant chemoradiotherapy in urethral squamous cell carcinoma. They also illustrate that single agent chemotherapy, namely, cisplatin, may be used successfully with limited toxicities.

## 1. Introduction

Primary urethral squamous cell carcinoma (SCC) is a rare entity accounting for less than 1% of all cancers [1]. Management of urethral cancer is a particularly challenging field owing to the paucity of evidence from randomised trials to inform practice. 

Historically, surgery and radiotherapy have been the main treatment modalities; however, significant morbidity may be conferred if an “R0” resection (complete resection with no microscopic residual tumor) is to be achieved [2]. Concomitant chemoradiotherapy has been considered by extrapolating evidence from other SCC's of the pelvis such as anal and cervical cancers [3–5]. 

We report two male and female cases of SCC of the urethra, which were treated successfully with concomitant cisplatin and radiotherapy.

## 2. Case  1

**Figure 1:**
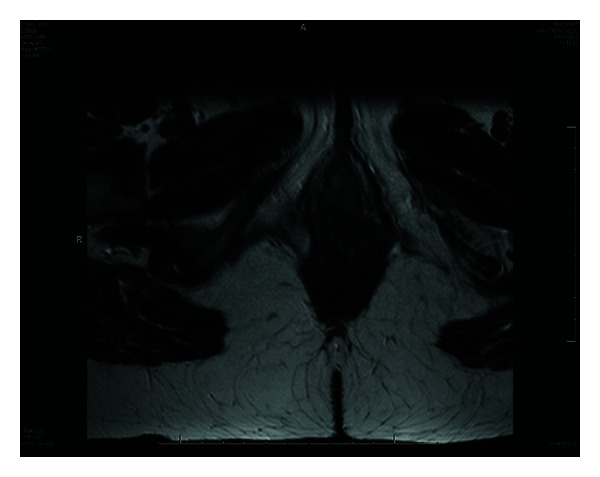


A 55-year-old lady presented with a history of intermittent per vaginal bleeding and haematuria in January 2011. Cystoscopy demonstrated a mobile solid, fleshy tumour at the external urethral meatus. Wedge biopsy confirmed a moderate to poorly differentiated distal urethral SCC, which invaded into the muscle. Magnetic resonance imaging (MRI) demonstrated malignant local invasion and possible extension into the perineum and vaginal orifice; see Figure 1. 

The patient underwent concomitant chemoradiotherapy with weekly cisplatin 40 mg m^−2^ for five weeks. Conformal radiotherapy using 50.4 Gray in 28 fractions was given in 2 phases over 5.5 weeks. Phase 1: 45 Gray in 25 fractions to the primary tumour and the regional lymph nodes and phase 2: 5.4 Gray in 3 fractions to the primary tumour. 

During the treatment, the patient experienced a grade 3 pelvic skin reaction and a grade 2 diarrhoea (common terminology criteria version (CTC) 3.0) [6]. Toxicity was managed supportively and did not interrupt treatment.

**Figure 2:**
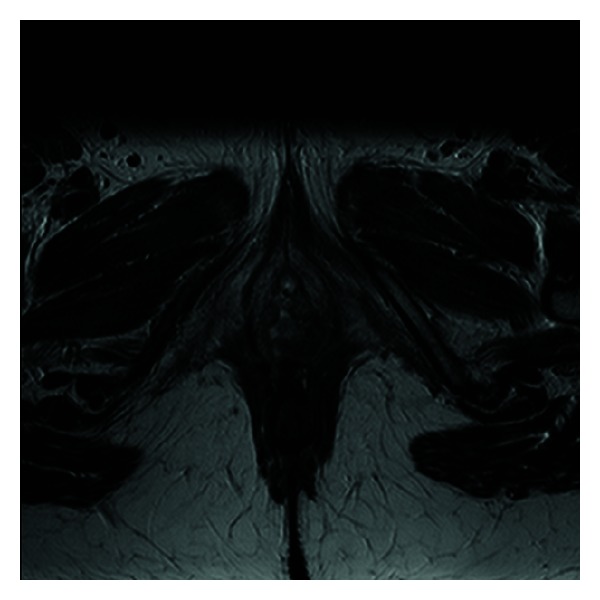


Posttreatment MRI demonstrated a complete response; see Figure 2. Repeat cystoscopy and biopsy at 4 months did not reveal any residual tumour. Two years, on the patient remains disease-free.

## 3. Case  2

**Figure 3:**
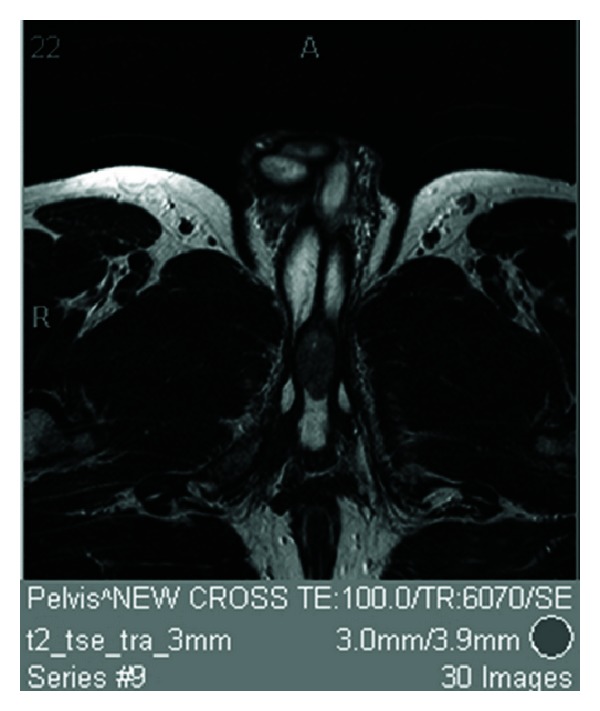


A 43-year-old man presented with a 2-month history of difficulty voiding urine and terminal dribbling. Cystourethroscopy demonstrated a polypoid growth in the bulbar urethra that prevented navigation to the bladder. A further cystourethroscopy under anaesthetic revealed only the polypoid tumour. There were no other abnormalities in the lower urinary tract. Subsequent biopsies confirmed a poorly to moderately differentiated polypoid SCC of the bulb of the urethra, which invaded into the stroma, at least stage pT2. Subsequent MRI scan revealed a 3.5 × 1.7 cm tumour invading into the proximal corpus spongiosum with some associated small inguinal nodes at 8 mm, which were not thought to be malignant. See Figure 3. 

Radical cystoprostatectomy was recommended, but the patient declined given the morbidity associated. Chemoradiotherapy treatment was therefore offered. A suprapubic catheter was sited due to the risk of obstruction from inflammation in the urethra during radiotherapy. Chemoradiotherapy with weekly cisplatin 40 mg m^−2^ for 6 weeks with 64 Gray in 32 fractions of conformal radiotherapy was given in 2 phases. Phase 1: 44 Gray in 22 fractions to the primary tumour with a margin of 2.5 cm craniocaudally and 1.5 cm circumferentially. Phase 2: 20 Gray in 10 fractions to the primary tumour with a geometric margin of 1.5 cm. 

CTC grade 3 pelvic skin toxicity was noted, this responded well to supportive measures. 

**Figure 4:**
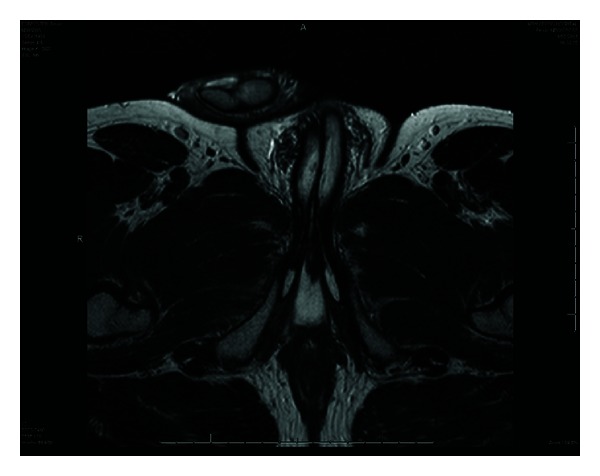


The suprapubic catheter was removed two months following completion of chemoradiotherapy. MRI at two months after treatment confirmed a complete response with low signal changes of the tumour sized at 2.2 × 0.8 cm, which was felt to be postradiotherapy change. The inguinal lymph nodes previously noted remained unchanged at 8 mm, supporting their benign nature. Urethroscopy was undertaken which confirmed scar tissue in the bulbar urethra. Biopsies were taken which did not show any evidence of residual tumour. See Figure 4. Eighteen months, on the patient remains disease free.

Both cases were discussed at the Urology Multi-Disciplinary Meeting where it was agreed that they represented primary urethral cancers rather than metastases from other pelvic structures.

## 4. Discussion

Urethral SCC is rare and can be challenging to manage due to the lack of randomised evidence to inform practice. Most patients present with locally advanced disease as urethral tumours tend to invade local structures such as bladder, prostate, or vagina. The above cases illustrate that urethral SCC can be successfully treated with concomitant cisplatin and radiotherapy in selected patients. 

In case one, a conventional conformal pelvic radiotherapy schedule of 50.4 Gray in 28 fractions was given due to the location of the tumour at the terminal urethra. Although testing for human papilloma virus (HPV) DNA or p16 was not undertaken, it was anticipated that this tumour would behave in a similar way to other anogenital SCCs that have an aetiological link to HPV such as anal cancer, and cancer of the uterine cervix [7–9]. Perinea toxicity from higher doses of radiotherapy would have been unacceptably high. Cisplatin as a single agent was used given its standard use in cervical cancer and using duel agent chemotherapy would likely incur increased toxicity. In case 2, 64 Gray in 32 fractions was given as the treatment volume was relatively small and anticipated to be well tolerated. 

Table 1 illustrates a summary of the published literature from PubMed spanning several decades and the modalities used [10–19].

In the last few decades, there has been a drive to establish genital preserving strategies for urethral squamous cell cancer. Intensity modulated radiotherapy (IMRT) is now standard practice for some tumour sites in most UK hospitals, for example, for head and neck cancer. IMRT allows a more conformal dose distribution and may allow for dose escalation to the primary tumour with better sparing of organs at risk such as bladder and rectum. 

Human papilloma virus (HPV) has been implicated in many types of SCC, conferring a better prognosis in head and neck in particular [20]. HPV has been implicated in both male and female urethral cancers, although with such rare cancers, it is unlikely to be able to help stratify patients into prognostic groups [21, 22].

These two cases add to the body of case reports that have shown benefit for chemoradiotherapy in urethral SCC. They also illustrate that single agent chemotherapy, namely, cisplatin, may be used successfully with limited toxicities. 

Randomised clinical trials remain the ultimate goal; however, with rare cancers, amalgamating experience is vital to inform practice.

## Figures and Tables

**Table 1:** Multimodality treatment of squamous cell carcinoma of the urethra.

Reference Year	*N *	Stage	Chemotherapy regime	Radiotherapy Dose (Gray)	Surgery	Outcome
[10] 1985	1	Locally advanced Female	Mitomycin C and 5-fluorouracil			Disease-free at 30 months

[11] 1989	1	Locally advanced SCC	Mitomycin C 15 mg/m^2^ and 5-fluorouracil 1250 mg/m^2^	40 Gy in 20 fractions		Disease-free at 28 months

[12] 1992	1	Locally advanced SCC Male	5-Fluorouracil 1 mg/m^2^ and mitomycin C 5 mg/m^2^	40 Gy in 20 fractions	Distal urethrectomy with en bloc resection of the adjacent corpora cavernosa	Cure

[13] 1995	4	Locally advanced SCC Male and female	5-Fluorouracil (100 mg/m^2^) and mitomycin C (15 mg/m^2^)	30–50 Gy		43–98 months median survival

[14] 1995	1	Locally advanced SCC Male	5-Fluorouracil mitomycin C			

[15] 1995	1	Stage IVB SCC	5-Fluorouracil 1400 mg and mitomycin C 18 mg	55.80 Gy in 31 fractions		Disease free at 5.5 yrs

[16] 1998	3	Locally advanced SCC	5-Fluorouracil and cisplatin	45 Gy	Distal urethrectomy	50% DFS

[17] 2004	2	Locally advanced SCC	5-Fluorouracil 750 mg/m^2^ and cisplatin 60 mg/m^2^	60 Gy in 30 fractions		One LR at 42 months The other is disease-free at 27 months

[18] 2008	18	T2N0 (11%) T3N0 (44%) T4N0 (11%) TXN1 (6%) TXN2 (28%)	5-Fluorouracil (1000 mg/m^2^) and mitomycin C (10 mg/m^2^)	45–55 Gy 25 fractions	Salvage surgery in selected cases	54% 5 year DFS chemoradiation 72% 5 year DFS chemoradiation and salvage surgery

[19] 2011	1	Locally advanced	Cisplatin weekly Following surgery: Adjuvant cisplatin and 5-fluorouracil Doses not available	60 Gy in 30 fractions	Salvage surgery 12 weeks after chemoradiotherapy due to local progressive disease: radical en bloc resection, abdominoperineal resection, right inguinal superficial lymphadenectomy, cystoprostatectomy with ileal conduit, penectomy, scrotal incision, and bilateral orchiectomy.	Symptom control for 5 months

*N*: number of patients, SCC: squamous cell carcinoma, DFS: disease-free survival, LR: Local recurrence, and Gy: Gray.

## References

[1] Eng TY, Naguib M, Galang T, Fuller CD (2003). Retrospective study of the treatment of urethral cancer. *American Journal of Clinical Oncology*.

[2] Swartz MA, Porter MP, Lin DW, Weiss NS (2006). Incidence of primary urethral carcinoma in the United States. *Urology*.

[3] (1996). Epidermoid anal cancer: results from the UKCCCR randomised trial of radiotherapy alone versus radiotherapy, 5-fluorouracil, and mitomycin. UKCCCR Anal Cancer Trial Working Party. UK Co-ordinating Committee on Cancer Research. *The Lancet*.

[4] Bartelink H, Roelofsen F, Eschwege F (1997). Concomitant radiotherapy and chemotherapy is superior to radiotherapy alone in the treatment of locally advanced anal cancer: results of a phase III randomized trial of the European Organization for Research and Treatment of Cancer Radiotherapy and Gastrointestinal Cooperative Groups. *Journal of Clinical Oncology*.

[5] Chemoradiotherapy for Cervical Cancer Meta-Analysis Collaboration (2008). Reducing uncertainties about the effects of chemoradiotherapy for cervical cancer: a systematic review and meta-analysis of individual patient data from 18 randomized trials. *Journal of Clinical Oncology*.

[6] DTCD N, NIH, DHHS

[7] Bosch FX, Lorincz A, Muñoz N, Meijer CJLM, Shah KV (2002). The causal relation between human papillomavirus and cervical cancer. *Journal of Clinical Pathology*.

[8] de Vuyst H, Clifford GM, Nascimento MC, Madeleine MM, Franceschi S (2009). Prevalence and type distribution of human papillomavirus in carcinoma and intraepithelial neoplasia of the vulva, vagina and anus: a meta-analysis. *International Journal of Cancer*.

[9] Zandberg DP, Bhargave R, Badin S, Cullen KJ (2013). The role of human papilloma virus in non-genital cancers. *CA: A Cancer Journal for Clinicians*.

[10] Shah AB, Kalra JK, Silber L, Molho L (1985). Squamous cell cancer of female urethra. Successful treatment with chemoradiotherapy. *Urology*.

[11] Johnson DW, Kessler JF, Ferrigni RG, Anderson JD (1989). Low dose combined chemotherapy/radiotherapy in the management of locally advanced urethral squamous cell carcinoma. *Journal of Urology*.

[12] Baskin LS, Turzan C (1992). Carcinoma of male urethra: management of locally advanced disease with combined chemotherapy, radiotherapy, and penile-preserving surgery. *Urology*.

[13] Licht MR, Klein EA, Bukowski R, Montie JE, Saxton JP (1995). Combination radiation and chemotherapy for the treatment of squamous cell carcinoma of the male and female urethra. *Journal of Urology*.

[14] Lutz ST, Huang DT (1995). Combined chemoradiotherapy for locally advanced squamous cell carcinoma of the bulbomembranous urethra: a case report. *Journal of Urology*.

[15] Tran LN, Krieg RM, Szabo RJ (1995). Combination chemotherapy and radiotherapy for a locally advanced squamous cell carcinoma of the urethra: a case report. *Journal of Urology*.

[16] Gheiler EL, Tefilli MV, Tiguert R, de Oliveira JG, Pontes JE, Wood DP (1998). Management of primary urethral cancer. *Urology*.

[17] Hara I, Hikosaka S, Eto H (2004). Successful treatment for squamous cell carcinoma of the female urethra with combined radio- and chemotherapy. *International Journal of Urology*.

[18] Cohen MS, Triaca V, Billmeyer B (2008). Coordinated chemoradiation therapy with genital preservation for the treatment of primary invasive carcinoma of the male urethra. *Journal of Urology*.

[19] Memon S, Craig Lynch L, Cleeve L, Murphy DG, Phol MJ, Heriot AG (2011). Squamous cell carcinoma of the bulbar urethra. *Journal of Clinical Oncology*.

[20] Ang KK, Harris J, Wheeler R (2010). Human papillomavirus and survival of patients with oropharyngeal cancer. *The New England Journal of Medicine*.

[21] Wiener JS, Liu ET, Walther PJ (1992). Oncogenic human papillomavirus type 16 is associated with squamous cell cancer of the male urethra. *Cancer Research*.

[22] Wiener JS, Walther PJ (1994). A high association of oncogenic human papillomaviruses with carcinomas of the female urethra: polymerase chain reaction-based analysis of multiple histological types. *Journal of Urology*.

